# Human mesenchymal stem cells

**DOI:** 10.1111/cpr.13141

**Published:** 2021-12-22

**Authors:** Xiaoyong Chen, Jing Huang, Jun Wu, Jie Hao, Boqiang Fu, Ying Wang, Bo Zhou, Tao Na, Jun Wei, Yong Zhang, Qiyuan Li, Shijun Hu, Jiaxi Zhou, Junying Yu, Zhaohui Wu, Huanxin Zhu, Jiani Cao, Lei Wang, Yaojin Peng, Lingmin Liang, Aijin Ma, Yu Zhang, Tongbiao Zhao, Andy Peng Xiang

**Affiliations:** ^1^ Center for Stem Cell Biology and Tissue Engineering Key Laboratory for Stem Cells and Tissue Engineering Ministry of Education Sun Yat‐Sen University Guangzhou China; ^2^ Chinese Society for Stem Cell Research Shanghai China; ^3^ National Stem Cell Resource Center State Key Laboratory of Stem Cell and Reproductive Biology Institute of Zoology Institute for Stem Cell and Regeneration Chinese Academy of Sciences Beijing China; ^4^ University of Chinese Academy of Sciences Beijing China; ^5^ Beijing Institute for Stem Cell and Regenerative Medicine Beijing China; ^6^ China National Institute of Metrology Beijing China; ^7^ Shanghai Institute of Nutrition and Health Chinese Academy of Sciences Shanghai China; ^8^ Shanghai Institute of Biochemistry and Cell Biology Chinese Academy of Sciences Shanghai China; ^9^ National Institutes for Food and Drug Control Beijing China; ^10^ Zephyrm Biotechnologies Co., Ltd Beijing China; ^11^ HHLIFE Company Inc. Shenzhen Shenzhen China; ^12^ China National GeneBank Shenzhen China; ^13^ Department of Cardiovascular Surgery of the First Affiliated Hospital & Institute for Cardiovascular Science Collaborative Innovation Center of Hematology State Key Laboratory of Radiation Medicine and Protection Medical College Soochow University Suzhou China; ^14^ Institute of Hematology and Blood Diseases Hospital Chinese Academy of Medical Sciences & Peking Union Medical College Tianjin China; ^15^ Nuwacell Biotechnology Co., Ltd Hefei City China; ^16^ China Medicinal Biotech Association Beijing China; ^17^ Beijing Technology and Business University Beijing China

## Abstract

Mesenchymal stem cells (MSCs) have attracted great interest for cell therapy and tissue regeneration due to their self‐renewal capacity, multipotency and potent immunomodulatory effects on immune cells. However, heterogeneity of MSCs has become a prominent obstacle to limit their translation into practice, as cells from different tissue sources or each individual have great differences in their transcriptomic signatures, differentiation potential and biological functions. Therefore, there is an urgent need for consensus standard for the quality control and technical specifications of MSCs. ‘Human Mesenchymal Stem Cells’ is the latest set of guidelines on hMSC in China, jointly drafted and agreed upon by experts from the Chinese Society for Stem Cell Research. This standard specifies the technical requirements, test methods, test regulations, instructions for use, labelling requirements, packaging requirements, storage requirements, transportation requirements and waste disposal requirements for hMSC, which is applicable to the quality control for hMSC. It was originally released by the China Society for Cell Biology on 9 January 2021. We hope that publication of these guidelines will facilitate institutional establishment, acceptance and execution of proper protocols, and accelerate the international standardization of hMSC for clinical development and therapeutic applications.

## 1 INTRODUCTION

Mesenchymal stem cells (MSCs) are multipotent stromal cells existing in many human tissues, and characterized by differentiating into mesodermal lineage cells. The term MSCs was coined by Caplan in 1991. Since Friedenstein and coworkers described the first isolation of MSCs from bone morrow, MSCs have been derived from lots of tissues. In 2006, the International Society for Cell Therapy (ISCT) proposed three criteria for the identification of MSCs: (1) demonstration of plastic‐adherent growth; (2) expression of CD105, CD73 and CD90; and a lack of expression of CD45, CD34, CD14 or CD11b, CD79a or CD19 and HLA‐DR and (3) demonstration of the ability to differentiate into osteoblasts, adipocytes, chondroblasts in vitro.

Mesenchymal stem cells have attracted great interest for cell therapy and tissue regeneration due to their self‐renewal capacity, multipotency and potent immunomodulatory effects on immune cells. However, heterogeneity of MSCs has become a prominent obstacle to limit their translation into practice, as cells from different tissue sources or each individual have great differences in their transcriptomic signatures, differentiation potential and biological functions. In addition, expansion protocols and passage number also associated with their functional properties. Therefore, it is important to consider establishing quality control and technical specifications for MSCs, not just their identification.

‘Human Mesenchymal Stem Cells’ is the guidelines on hMSC in China, jointly drafted and agreed upon by experts from the Chinese Society for Stem Cell Research. This consensus standard for MSCs was established based on their basic biological characteristics, microbiological safety, biological safety and biofunctional properties This standard specifies the technical requirements, test methods, test regulations, instructions for use, labelling requirements, packaging requirements, storage requirements, transportation requirements and waste disposal requirements for human mesenchymal stem cells (hMSC), which is applicable to the quality control for hMSC. The guideline of MSCs has been originally released by the China Society for Cell Biology on 9 January 2021. Now it is translated into an English version. We hope that publication of these guidelines will facilitate institutional establishment, acceptance and execution of proper protocols, and accelerate the international standardization of hMSC for clinical development and therapeutic applications.

## 2 HUMAN MESENCHYMAL STEM CELLS STANDARD

### 2.1 Scope

This document specifies the technical requirements for hMSC, and requirements for test methods, test regulations, instructions for use, labelling, packaging, storage, transportation and waste disposal.

This standard is applicable for the production and testing of hMSC.

### 2.2 Normative references

The following content constitute indispensable articles of this standard through normative reference. For dated references, only the edition cited applies. For undated references, only the latest edition (including all amendments) applies.

GB/T 6682 *Water for analytical laboratory use – Specification and test methods*.

WS213 *Diagnosis for hepatitis C*.

WS273 *Diagnosis for syphilis*.

WS293 *Diagnosis for HIV/AIDS*.

T/CSCB 0001‐2020 *General requirements for stem cells*.

*Pharmacopoeia* *of the People's Republic of China*.

*National* *Guide to Clinical Laboratory Procedures*.

### 2.3 Terms and definitions

For the purpose of this document, the terms and definitions in T/CSCB 0001, T/CSCB 0002 and following terms and definitions apply to this document.

#### 2.3.1 Human mesenchymal stem cells

A type of stem cells exhibit a fibroblast‐like morphology (spindle and fusiform) after adherent culture, and possess the ability to self‐renew and differentiate into the osteogenic, adipogenic and chondrogenic lineages in vitro.

Note 1 to entry: hMSC can not only be isolated from multiple tissues (such as bone marrow, umbilical cord, placenta, adipose tissue, umbilical cord blood et al) but also be obtained by differentiation or transdifferentiation. hMSC from different sources have differences in gene expression profile and differentiation potential.

### 2.4 Abbreviations

The following abbreviations are applicable for this document.

CD: cluster of differentiation.

DNA: deoxyribonucleic acid.

EBV: Epstein – Barr virus.

HBV: hepatitis B virus.

HCMV: human cytomegalovirus.

HCV: hepatitis C virus.

HIV: human immunodeficiency virus.

HLA‐DR: human leukocyte antigen‐DR.

HTLV: human T‐lymphotropic virus.

IDO: indoleamine 2, 3‐dioxygenase.

IFN‐γ: interferon gamma.

TNF‐α: tumour necrosis factor alpha.

PCR: polymerase chain reaction.

STR: short tandem repeat.

TP: treponema pallidum.

### 2.5 Technical requirements

#### 2.5.1 Source materials and ancillary materials

1. The source materials, reagents, consumables and other ancillary materials and/or supplies (eg gases) shall meet the requirements of T/CSCB 0001.
2. To ensure the safety of the donor and the donated cells, the process for donor evaluation and screening, cell collection, transportation and receipt shall be standardized.
3. Donors shall be screened for HIV, HBV, HCV, HTLV, EBV, HCMV, TP and the results shall be documented.

#### 2.5.2 Primary quality attributes

1. Cell morphology

Cells under adherent culture shall exhibit a fibroblast‐like morphology (spindle and fusiform), and uniform morphology.

2. Chromosome karyotype

The normal karyotype shall be 46, XX or 46, XY.

3. Cell viability

The cell viability shall be ≥90% prior to cryopreservation, and ≥70% after thawing.

4. Cell surface markers

The expressions of CD105, CD73 and CD90 shall be ≥95% of the cell population, and the expression of CD11b, CD19, CD31, CD34, CD45 and HLA‐DR shall be ≤2% of the cell population.

5. Immunomodulatory capacity

Cells shall be induced to express indoleamine 2,3‐dioxygenase (IDO) with the treatment of inflammatory factors (IFN‐γ or IFN‐γ + TNF‐α). Additionally, after co‐cultured with T cells, these cells shall broadly suppress T cell proliferation and the secretion of IFN‐γ and TNF‐α.

6. Tri‐lineage differentiation capacity

Cells shall be able to differentiate to osteoblasts, adipocytes and chondroblasts under standardized differentiating conditions in vitro.

7. Tumorigenesis

Cells shall be negative for in vivo tumorigenicity testing using immunodeficient animal model (such as immunodeficient mice).

8. Microorganisms

Cells shall be negative for fungi, bacteria, mycoplasma, HIV, HBV, HCV, HTLV, EBV, HCMV and TP.

#### 2.5.3 Process control

1. The process of cell expansion, cryopreservation, cell thawing and other cell manufacturing shall follow the requirements of T/CSCB 0001‐2020.
2. The STR test results of cell products shall be consistent with STR of the donor cell.

### 2.6 Test methods

#### 2.6.1 Cell morphology

Observe the morphology of cells grown in 2D condition using a microscope.

#### 2.6.2 Chromosome karyotype

The method in the *Pharmacopoeia of the People's Republic of China* shall be followed.

#### 2.6.3 Cell viability

The method in Appendix A shall be followed.

#### 2.6.4 Cell surface markers

The method in Appendix B shall be followed.

#### 2.6.5 Immunomodulatory capacity

1. The induced expression of IDO

The method in Appendix C shall be followed.

2. The suppression of T cell proliferation

The method in Appendix D shall be followed.

3. The suppression of secretion of IFN‐γ and TNF‐α by T cells

The method in Appendix E shall be followed.

#### 2.6.6 Tri‐lineage differentiation capacity

1. Osteogenic differentiation

The method in Appendix F shall be followed.

2. Adipogenic differentiation

The method in Appendix G shall be followed.

3. Chondrogenic differentiation

The method in Appendix H shall be followed.

#### 2.6.7 Tumorigenesis

The method in Appendix I shall be followed.

#### 2.6.8 Microorganisms

1. Fungi

The ‘1101 Sterility Inspection Method’ in *Pharmacopoeia of the People's Republic of China* (Volume IV) shall be followed.

2. Bacteria

The ‘1101 Sterility Inspection Method’ in *Pharmacopoeia of the People's Republic of China* (Volume IV) shall be followed.

3. Mycoplasma

The ‘3301 Mycoplasma Inspection Method’ in *Pharmacopoeia of the People's Republic of China* (Volume IV) shall be followed.

4. HBV

The method in *National Guide to Clinical Laboratory Procedures* shall be followed.

5. HCV

The method in WS 293 shall be followed.

6. HIV

The method in WS 293 shall be followed.

7. HTLV

The method in *National Guide to Clinical Laboratory Procedures* shall be followed.

8. EBV

The method in *National Guide to Clinical Laboratory Procedures* shall be followed.

9. HCMV

The method in *National Guide to Clinical Laboratory Procedures* shall be followed.

10. TP

The method in WS 293 shall be followed.

### 2.7 Inspection rules

#### 2.7.1 Sampling method

1. Cells produced from the same production cycle, same production line, same source, same passage and same method are considered to be the same batch.
2. Three smallest units of packaging shall be randomly sampled from the same batch.

#### 2.7.2 Quality inspection and release

1. Each batch of products shall be subject to the qualify inspection before release, and inspection reports shall be attached.
2. The quality inspection items shall include all the attributes specified in 2.5.2.

#### 2.7.3 Review inspection

Review inspection shall be performed by professional cytological testing institutions or laboratories as necessary.

#### 2.7.4 Decision rules

1. Products that pass all requirements in 2.5.2 for the quality inspection for release are considered to be qualified. Products that fail to pass one or more requirements in 2.5.2 for the quality inspection for release are considered to be unqualified.
2. Products that pass all requirements in 2.5.2 for the quality review inspection are considered to be qualified. Products that fail to pass one or more requirements in 2.5.2 for the review inspection are considered to be unqualified.

### 2.8 Instructions for usage

The instructions for usage shall include, but not limited to:

- Product name;
- Passage number;
- Cell numbers;
- Production date;
- Lot number;
- Production organization;
- Storage conditions;
- Shipping conditions;
- Operation manual;
- Execution standard number;
- Manufacturing address;
- Contact information;
- Postal code;
- Matters that need attention.

Note 1 to entry: Endotoxin content can be listed according to user needs.

### 2.9 Labels

The label shall include but not limited to:

- Product name;
- Passage number;
- Cell number;
- Lot number;
- Production organization;
- Production date.

### 2.10 Package, storage and transportation

#### 2.10.1 Package

The appropriate materials and containers shall be selected to ensure maintenance of the primary quality attributes of hMSC.

#### 2.10.2 Storage

1. T/CSCB 0001‐2020 shall be followed.
2. Cell products shall be stored at a temperature below −130℃.

#### 2.10.3 Transportation

1. T/CSCB 0001‐2020 shall be followed.
2. Cryopreserved cell products shall be transported in dry ice or at a temperature below −130℃. Non‐frozen cells are recommended for refrigerated transportation (2℃ ~ 8℃).

### 2.11 Waste disposal

The waste generated during manufacturing and testing of hMSC shall be disposed following the requirements of T/CSCB 0001‐2020.

## CONFLICT OF INTEREST

No potential conflicts of interest are disclosed.

## AUTHOR CONTRIBUTION

Xiang AP, Zhao T, Zhang Y and Ma A contributed to conception and design. Chen X, Huang J, Wu J and Hao J drafted and revised the manuscript. Fu B, Wang Y, Zhou B, Na T, Wei J, Zhang Y, Li Q, Hu S, Zhou J, Yu J, Wu Z, Zhu H, Cao J, Wang L, Peng Y and Liang L critically read and revised the manuscript.

## Supporting information


**Appendix A:** Cell viability test (cell enumeration method)
**Appendix B:** Detection of cell surface markers (Flow cytometry)
**Appendix C:** Detection of induced IDO expression (PCR assay)
**Appendix D:** T cell proliferation inhibition assay (CFSE assay)
**Appendix E:** Inhibition assay of IFN‐γ and TNF‐α secretion by T cells (Intracellular Cytokine Staining, ICS)
**Appendix F:** Osteogenic differentiation assay (Alizarin red S staining)
**Appendix G:** Adipogenic differentiation assay (Oil red O staining)
**Appendix H:** Chondrogenic differentiation assay (Alcian blue staining)
**Appendix I:** In vivo Tumorigenicity Testing (immunodeficient mice method)

## Data Availability

The authors confirm that the data supporting the findings of this study are available within the article.

## APPENDIX A

### Normative appendix: Cell viability test (cell enumeration method)

**A.1 Instruments**

A.1.1 Microscope.

A.1.2 Haemocytometer.

**A.2 Reagents**

Unless otherwise stated, all reagents used shall be analytical grade. The water used for testing shall be deionized water.

A.2.1 Phosphate‐buffered saline (PBS): pH 7.4.

A.2.2 Trypan blue solution: dilute to 0.4% (W/V) with phosphate‐buffered saline (A.2.1).

**A.3 Testing protocol**

A.3.1 Preparation of cell suspension

Harvest and suspend the cells with appropriate volume of Phosphate‐buffered saline (A.2.1). The cells in the haemocytometer shall be 20 ~ 50 cells/mm^2^. Serial dilution is necessary if the number of cells exceeds 200 per haemocytometer.

A.3.2 Trypan blue staining

Evenly mix the Trypan blue solution (A.2.2) with the cell suspension (A.3.1) at a volume ratio of 1:1.

A.3.3 Cell counting

Load the haemocytometer (A.1.2) with 10 μL of the trypan blue‐labelled sample (A.3.2). Make sure the entire chamber is filled with the testing sample. Stand for 30 seconds, count the stained cells and the total number of cells respectively.
For the 16 × 25 counting chamber, use the four 1 mm^2^ medium squares at the top left, top right, bottom left and bottom right of the chamber (ie 100 small squares) for counting. For the 25 × 16 counting chamber, use the five 1 mm^2^ medium squares at the top left, top right, bottom left, bottom right and centre of the chamber (i.e. 80 small squares) for counting. When there are cells on the lines of the large square, only cells on the top line and left line of the large square can be counted (or alternatively only cells on the bottom line and right line).
Repeat steps A.3.2 ~ A.3.3 for another sample.

A.3.4 Cell survival rate calculation

**A.4 Calculation and analysis**

Cell viability is calculated according to equation (A1)

(A1)
$$\text{Cell viability}=\left(M‐S\right)/M\times 100\%$$

In this equation: M—total number of cells. S—number of stained cells.
The viability of cells is the mean of two duplicate samples.

**A.5 Accuracy**

The absolute difference value between the two independent tests by the independent inter‐examiner, under the same conditions, shall not exceed 10% of their arithmetic mean.

## APPENDIX B

### Normative appendix: Detection of cell surface markers (Flow cytometry)

**B.1 Instruments**

B.1.1 Flow cytometer.

B.1.2 Bench‐top centrifuge.

B.1.3 Electronic balance.

**B.2 Reagents**

Unless otherwise stated, all the reagents used shall be analytical grade. The water used in the experiment shall be Grade 1 water as stipulated in GB/T 6682.

B.2.1 Phosphate‐buffered saline (PBS): pH7.4.

B.2.2 Bovine serum albumin (BSA): Purity ≥98%.

B.2.3 Sodium azide (NaN3).

B.2.4 Antibodies (including Anti‐human CD105, CD73, CD90, CD11b, CD19, CD31, CD34, CD45, HLA‐DR antibodies and isotype control antibodies).

Note 1 to entry: Make sure all the antibodies work well according to the manufacturer’s instructions. Live or dead cell staining could be used where necessary.

B.2.5 Use electronic balance (B.1.3), prepare the following solutions according to the relative requirements for flow cytometry: wash solution, antibody dilution solution.

**B.3 Sample storage**

The wash solution and fixed samples shall be stored at 2 ~ 8°C. Antibodies shall be stored according to the manufacturer’s instructions.

**B.4 Testing protocol**

B.4.1 Sample preparation

Collect samples by centrifuging single‐cell suspensions with bench‐top centrifuge (B.1.2) at 300 g for 4 min and discard the supernatant. Wash the cell samples with an appropriate volume of wash solution, then collect samples by centrifuging at 300 g for 4 min and discard the supernatant.

B.4.2 Antibody incubation

Incubate the samples with the diluted antibodies according to the manufacturer’s instructions. Wash the cell samples with an appropriate volume of wash solution for 2 times, then centrifuge at 300 g for 4 min and discard the supernatant.

B.4.3 Filter and detection

Resuspend the samples with wash solution and then transfer the cell suspension into flow cytometry tube passed through a 40µm mesh filter. Load the samples into the flow cytometer and perform detection according to the manufacturer’s instruction.

B.4.4 Gating

Gate the population of target cells based on particle size and granularity, excluding cell debris and other irrelevant particles. The gating of positive staining cells shall be determined by the fluorescence intensity using isotype controls as a reference. Both positive and negative experimental controls shall be set up for gating and the following analysis.

**B.5 Analysis of results**

Analyse the results using software according to manufacturer’s instructions.

## APPENDIX C

### Normative appendix: Detection of induced IDO expression (PCR assay)

**C.1 Instruments**

C.1.1 Nucleic acid quantification machine.

C.1.2 PCR‐Cycler.

C.1.3 Electrophoresis apparatus

C.1.4 Gel imager

**C.2 Reagents**

Unless otherwise stated, all the reagents used shall be analytical grade. The water used in the experiment shall be Grade 1 water as stipulated in GB/T 6682.

C.2.1 Human recombinant IFN‐γ and human recombinant TNF‐α.

C.2.2 RNA extraction kit.

C.2.3 Reverse transcription kit.

C.2.4 PCR primers.

C.2.5 Taq DNA polymerase.

C.2.6 Ladders.

**C.3 Testing protocol**

C.3.1 Treat the human mesenchymal stem cells with IFN‐*γ* or IFN‐*γ* + TNF‐*α*

Calculate the living cell concentration of human mesenchymal stem cells (hMSC) suspension according to the method in Appendix A. Seed hMSC at a density of 1 × 10^5^ ~ 4 × 10^5^ cells/cm^2^, then culture with IFN‐γ (10 ~ 30 ng/mL) or IFN‐γ (10 ~ 30 ng/mL) + TNF‐α (10 ~ 30 ng/mL), for 12 ~ 36 h. At the same time, hMSC culture without IFN‐γ or IFN‐γ + TNF‐α are set up as the control group and cultured for the same time.

C.3.2 Extraction of cellular RNA

Perform RNA extraction according to the manufacturer’s instructions and use a nucleic acid quantification machine (C.1.1) for nucleic acid content determination.

C.3.3 Reverse transcription‐polymerase chain reaction (RT‐PCR)

Perform reverse transcription to acquire cDNA and amplify the gene of IDO via PCR‐Cycler (C.1.2) according to the manufacturer’s instructions.

C.3.4 Electrophoresis of PCR products

Use electrophoresis apparatus (C.1.3) for electrophoresis testing that shall be performed according to the manufacturer’s instructions.

C.3.5 Gel imaging.

Perform the Gel imaging via Gel imager (C.1.4) according to the manufacturer’s instructions.

**C.4 Analysis of results**

The expression of IDO is detected in hMSC cultured with IFN‐γ or IFN‐γ + TNF‐α, while it is undetectable in hMSC without stimulation.

## APPENDIX D

### Normative appendix: T‐cell proliferation inhibition assay (CFSE assay)

**D.1 Instruments**

D.1.1 Haemocytometer.

D.1.2 Microscope.

D.1.3 Bench‐top centrifuge.

D.1.4 Flow cytometer.

**D.2 Reagents**

Unless otherwise stated, all the reagents used shall be analytical grade. The water used in the experiment shall be Grade 1 water as stipulated in GB/T 6682.

D.2.1 Phosphate‐buffered saline (PBS): pH7.4.

D.2.2 Cell dissociation enzyme.

D.2.3 Trypan Blue Solution: dilute to 0.4% (W/V) with phosphate‐buffered saline (D.2.1).

D.2.4 Phytohaemagglutinin, PHA

D.2.5 Leukocyte separation solution (Ficoll)

D.2.6 Antibodies (such as anti‐CD3 antibody)

D.2.7 Carboxyfluorescein diacetate succinimidyl ester (CFSE)

**D.3 Testing protocol**

D.3.1 T‐cell separation and staining

D.3.1.1 T‐cell separation

Use Ficoll (D.2.5) to separate peripheral blood mononuclear cells (PBMC) and wash twice with an appropriate volume of sterile phosphate‐buffered saline (D.2.1) by bench‐top centrifuge (D.1.3). Incubate the samples with the diluted antibodies and then wash twice with an appropriate volume of sterile phosphate‐buffered saline (D.2.1) by bench‐top centrifuge (D.1.3). Resuspend the samples and dilute the cell suspension to 5 × 10^7^ cells/mL with wash solution and then transfer the cell suspension into flow cytometry tube passed through a 40µm mesh filter. Load the samples into the flow cytometer (D.1.4) and perform cell sorting according to the manufacturer’s instructions.

D.3.1.2 T‐cell staining

Use haemocytometer (D.1.1) and microscope (D.1.2) to calculate the living cell concentration of T cells according to the method in Appendix A. Label T cells with CFSE according to the manufacturer’s instructions.

D.3.2 Co‐culture T cells with human mesenchymal stem cells

D.3.2.1 T‐cell proliferation

Use haemocytometer (D.1.1) and microscope (D.1.2) to calculate the living cell concentration of CFSE‐labelled T‐cell suspension according to the method in Appendix A. Seed the T cells at a density of 1 × 10^6^ cells/cm^2^ and stimulate with 2 ~ 5 µg/mL PHA (D.2.4). T cells culture without PHA are set up as the control group. After 96 h culture, the percentage of T‐cell proliferation after stimulation is detected and record it as A.

D.3.2.2 Suppression of T‐cell proliferation by human mesenchymal stem cells

Use cell dissociation enzyme to dissociate human mesenchymal stem cells (hMSC) and prepare as single‐cell suspension. Use haemocytometer (D.1.1) and microscope (D.1.2) to calculate the living cell concentration of hMSC suspension according to the method in Appendix A. Seed the hMSC at a density of 2 × 10^5^ cells/cm^2^. Then, co‐culture with CFSE‐labelled T cells at a ratio of 5:1 (T cells: hMSC) in the present of 2 ~ 5 µg/mL PHA (D.2.4). After 96 h culture, the percentage of T‐cell proliferation that cultured with hMSC is detected and record it as C.

D.3.3 Collection and detection of T cells

Collect the T cells (D.3.2) and wash T‐cell samples twice with sterile phosphate‐buffered saline (D.2.1) by bench‐top centrifuge (D.1.3), then transfer the cell suspension into flow cytometry tube passed through a 40 µm mesh filter. Load the samples into the flow cytometer (D.1.4) and perform detection according to the manufacturer’s instructions.

D.3.4 Gating

Gate the population 1 of target cells based on particle size and granularity, excluding cell debris and other irrelevant particles. Then, gate the parent population (zeroth generation) in population1 according to the fluorescence intensity of T cells without PHA stimulation and gate the proliferating cells assigned as population 2 (the percentage of T‐cell proliferation) based on the position of parent population.

**D.4 Analysis of results**

Analyse the results using software according to manufacturer’s instructions and calculate the inhibition rate of hMSC on T‐cell proliferation.
Inhibition rate is calculated according to equation (D1):

(D1)
$$\text{Inhibition rate}=\left(A‐C\right)/A\times 100\%$$

In this equation: A—the percentage of the proliferating T cells without hMSC. C—the percentage of the proliferating T cells that cultured with hMSC.

## APPENDIX E

### Normative appendix: Inhibition assay of IFN‐γ and TNF‐α secretion by T cells (Intracellular Cytokine Staining, ICS)

**E.1 Instruments**

E.1.1 Haemocytometer.

E.1.2 Microscope.

E.1.3 Bench‐top centrifuge.

E.1.4 Flow cytometer.

**E.2 Reagents**

Unless otherwise stated, all the reagents used shall be analytical grade. The water used in the experiment shall be Grade 1 water as stipulated in GB/T 6682.

E.2.1 Phosphate‐buffered saline (PBS): pH7.4.

E.2.2 Cell dissociation enzyme.

E.2.3 Trypan Blue Solution: dilute to 0.4% (W/V) with phosphate‐buffered saline (E.2.1).

E.2.4 Phorbol ester (PMA).

E.2.5 Leukocyte separation solution (Ficoll).

E.2.6 Antibodies (such as anti‐CD3, anti‐IFN‐γ and anti‐TNF‐α antibodies).

E.2.7 Brefeldin A (BFA).

E.2.8 Ionomycin.

E.2.9 Saponin.

E.2.10 4% PFA.

**E.3 Testing protocol**

E.3.1 T‐cell separation

Use Ficoll (E.2.5) to separate peripheral blood mononuclear cells (PBMC) and wash twice with an appropriate volume of sterile phosphate‐buffered saline (E.2.1) by bench‐top centrifuge (E.1.3). Incubate the PBMC with the diluted antibodies (E.2.6) and then wash twice with an appropriate volume of sterile phosphate‐buffered saline (H.2.1). Resuspend the PBMC and dilute the cell suspension to 5 × 10^7^ cells/mL with wash solution and then transfer the cell suspension into flow cytometry tube passed through a 40 µm mesh filter. Load the samples into the flow cytometer (E.1.4) and perform T‐cell sorting according to the manufacturer’s instructions.

E.3.2 Co‐culturing of T cells with human mesenchymal stem cells

E.3.2.1 T‐cell inflammatory factor secretion

Use haemocytometer (E.1.1) and microscope (E.1.2) to calculate the living cell concentration of T‐cell suspension according to the method in Appendix A. Seed T cells at a density of 1 × 10^6^ cells/cm^2^ and culture for 48 h. Add 50 ng/mL PMA (E.2.4), 1 μg/mL ionomycin (E.2.8) and 10 μg/mL BFA (E.2.7) to the culture system 4 ~ 6 h before the end of the culture. Detect the proportion of IFN‐γ^+^ T cells and record as A1, and the proportion of TNF‐α^+^T cells and record as A2.

E.3.2.2 Suppression T‐cell cytokine secretion by human mesenchymal stem cells

Use cell dissociation enzyme to dissociate human mesenchymal stem cells (hMSC) and prepare as single‐cell suspension. Use haemocytometer (E.1.1) and microscope (E.1.2) to calculate the living cell concentration of h hMSC and T‐cell suspension according to the method in Appendix A. Seed hMSC at a density of 2 × 10^5^ cells/cm^2^. Then, co‐culture with T cells at a ratio of 5:1 (T cells: hMSC). Culture for 48 h and add 50 ng/mL PMA (E.2.4), 1 μg/mL ionomycin (E.2.8) and 10 μg/mL BFA (E.2.7) to the culture system 4 ~ 6 h before the end of the culture. Detect the proportion of IFN‐γ^+^ T cells in the T/hMSC culture system and record as C1, and the proportion of TNF‐α^+^T cells in the T/hMSC culture system and record as C2.

E.3.3 Collection and detection of T cells

Collect the T cells (E.3.2) and wash T‐cell samples twice with sterile phosphate‐buffered saline (E.2.1) and fix cells with 4% PFA (E.2.10), then wash T‐cell samples twice with sterile phosphate‐buffered saline (E.2.1), and treat cells with 0.1% ~ 0.2% saponin (E.2.9). Incubate the samples with the diluted antibodies and wash T‐cell samples with sterile phosphate‐buffered saline (E.2.1), and transfer the cell suspension into flow cytometry tube passed through a 40 µm mesh filter. Load the samples into the flow cytometer (E.1.4) and perform detecting according to the manufacturer’s instructions.

E.3.4 Gating

Gate the population 1 of target cells based on particle size and granularity, excluding cell debris and other irrelevant particles. According to the fluorescence intensity of the isotype control, gate the cell population of IFN‐γ^+^ T cells within population 1, as well as the cell population of TNF‐α^+^ T cells, excluding negative cells that are not labelled by fluorescent antibodies.

**E.4 Analysis of results**

Analyse the results using software according to manufacturer’s instructions and calculate the inhibition rate of hMSC on the IFN‐γ and TNF‐α secretion of T cells.
Inhibition rate is calculated according to equation (E1) and (E2):

(E1)
$$\text{IFN}‐\gamma \;\text{inhibition rate}=\left(A1‐C1\right)/A1\times 100\%$$

In this equation: A1—the percentage of IFN‐γ^+^ T cells without hMSC. C1—the percentage of IFN‐γ^+^ T cells that cultured with hMSC.

(E2)
$$\text{TNF}‐\alpha \;\text{inhibition rate}=\left(A2‐C2\right)/A2\times 100\%$$

In this equation: A2—the percentage of TNF‐α^+^ T cells without hMSC. C2—the percentage of TNF‐α^+^ T cells that cultured with hMSC.

## APPENDIX F

### Normative appendix: Osteogenic differentiation assay (Alizarin red S staining)

**F.1 Instruments**

F.1.1 Haemocytometer.

F.1.2 Microscope.

F.1.3 Bench‐top centrifuge.

**F.2 Reagents**

Unless otherwise stated, all the reagents used shall be analytical grade. The water used in the experiment shall be Grade 1 water as stipulated in GB/T 6682.

F.2.1 Phosphate‐buffered saline (PBS): pH7.4.

F.2.2 Cell dissociation enzyme.

F.2.3 Trypan Blue Solution: dilute to 0.4% (W/V) with phosphate‐buffered saline (F.2.1).

F.2.4 Osteogenic differentiation medium.

F.2.5 Alizarin red S staining kit.

**F.3 Testing protocol**

F.3.1 Sample preparation

F.3.1.1 Cell dissociation

Use cell dissociation enzyme to dissociate human mesenchymal stem cells (hMSC), collect hMSC by bench‐top centrifuge (F.1.3) and gently resuspend the cells in saline to avoid the formation of bubbles or residual cell clumps.

F.3.1.2 Cell counting

Use haemocytometer (F.1.1) and microscope (F.1.2) to calculate the live cell concentration of the cell suspension, according to the method in Appendix A.

F.3.2 Cell seeding and induction

Choose the appropriate cell seeding method, seeding density and induction procedure according to the manufacturer’s instructions, and osteogenic differentiation is induced for 14 ~ 21 days.

F.3.3 Calcium deposits staining

Stain the extracellular calcium deposits by Alizarin Red S staining according to the manufacturer’s instructions.

**F.4** **Analysis of results**

A large number of bright orange‐red calcium deposits can be seen under the microscope.

## APPENDIX G

Normative appendix: Adipogenic differentiation assay (Oil red O staining)

**G.1 Instruments**

G.1.1 Haemocytometer.

G.1.2 Microscope.

G.1.3 Bench‐top centrifuge.

**G.2 Reagents**

Unless otherwise stated, all the reagents used shall be analytical grade. The water used in the experiment shall be Grade 1 water as stipulated in GB/T 6682.

G.2.1 Phosphate‐buffered saline (PBS): pH7.4.

G.2.2 Cell dissociation enzyme.

G.2.3 Trypan Blue Solution: dilute to 0.4% (W/V) with phosphate‐buffered saline (G.2.1).

G.2.4 Adipogenic differentiation medium.

G.2.5 Oil red O staining kit.

**G.3 Testing protocol**

G.3.1 Sample preparation

G.3.1.1 Cell dissociation

Use cell dissociation enzyme to dissociate human mesenchymal stem cells (hMSC), collect hMSC by bench‐top centrifuge (G.1.3) and gently resuspend the cells in saline to avoid the formation of bubbles or residual cell clumps.

G.3.1.2 Cell counting

Use haemocytometer (G.1.1) and microscope (G.1.2) to calculate the live cell concentration of the cell suspension, according to the method in Appendix A.

G.3.2 Cell seeding and induction

Choose the appropriate cell seeding method, seeding density and induction procedure according to the manufacturer’s instructions, and adipogenic differentiation is induced for 14 ~ 21 days.

G.3.3 Lipid droplet staining

Stain the lipid droplets by Oil red O according to the manufacturer’s instructions.

**G.4 Analysis of results**

Orange‐red lipid droplets can be seen under the microscope, and fat cells contain lipid droplets of varying sizes.

## APPENDIX H

### Normative appendix: Chondrogenic differentiation assay (Alcian blue staining)

**H.1 Instruments**

H.1.1 Haemocytometer.

H.1.2 Microscope.

H.1.3 Bench‐top centrifuge.

**H.2 Reagents**

Unless otherwise stated, all the reagents used shall be analytical grade. The water used in the experiment shall be Grade 1 water as stipulated in GB/T 6682.

H.2.1 Phosphate‐buffered saline (PBS): pH7.4.

H.2.2 Cell dissociation enzyme.

H.2.3 Trypan Blue Solution: dilute to 0.4% (W/V) with phosphate‐buffered saline (H.2.1).

H.2.4 Chondrogenic differentiation medium.

H.2.5 Alcian blue staining kit.

**H.3 Testing protocol**

H.3.1 Sample preparation

H.3.1.1 Cell dissociation

Use cell dissociation enzyme to dissociate human mesenchymal stem cells (hMSC), collect hMSC by bench‐top centrifuge (H.1.3) and gently resuspend the cells in saline to avoid the formation of bubbles or residual cell clumps.

H.3.1.2 Cell counting

Use haemocytometer (H.1.1) and microscope (H.1.2) to calculate the live cell concentration of the cell suspension, according to the method in Appendix A.

H.3.2 Cell seeding and induction

Choose the appropriate cell seeding method, seeding density and induction procedure according to the manufacturer’s instructions, and chondrogenic differentiation is induced for 14 ~ 21 days.

H.3.3 Cartilage extracellular matrix staining

Stain the cartilage extracellular matrix by Alcian blue according to the manufacturer’s instructions.

**H.4 Analysis of results**

The dark blue extracellular matrix of chondrocytes can be seen under the microscope.

## APPENDIX I

### Normative appendix: In vivo Tumorigenicity Testing (immunodeficient mice method)

**I.1 Instruments**

I.1.1 Haemocytometer.

I.1.2 Microscope.

I.1.3 Bench‐top centrifuge.

**I.2 Reagents**

Unless otherwise stated, all the reagents used shall be analytical grade. The water used in the experiment shall be Grade 1 water as stipulated in GB/T 6682.

I.2.1 Phosphate‐buffered saline (PBS): pH7.4.

I.2.2 Cell dissociation enzyme.

I.2.3 Trypan Blue Solution: dilute to 0.4% (W/V) with phosphate‐buffered saline (I.2.1).

**I.3 Testing protocol**

I.3.1 Sample preparation

I.3.1.1 Cell dissociation

Use cell dissociation enzyme to dissociate human mesenchymal stem cells (hMSC), collect hMSC by bench‐top centrifuge (I.1.3) and gently resuspend the cells in saline to avoid the formation of bubbles or residual cell clumps.

I.3.1.2 Cell counting

Use haemocytometer (I.1.1) and microscope (I.1.2) to calculate the live cell concentration of the cell suspension, according to the method in Appendix A.

I.3.2 Cell transplantation

1 × 10^7^ human mesenchymal stem cells are injected subcutaneously into immunodeficient mice aged 6–8 weeks. Set up a blank control group (injected with the solvent corresponding to the product), a negative control group (human diploid cells) and a positive control group (human tumour cell lines, injected according to the number of tumour cell line inoculation requirements).

I.3.3 Tumour observation

Observe 16 weeks after injection and measure body weight and tumour size every week. If the tumour in the tumour‐bearing mice exceeded 2000 mm^3^, the tumour was ulcerated or severe weight loss, the humane endpoint could be considered. Remove the tumour from the mice, and perform gross observation. Then, weigh the tumour and calculate the tumour formation rate.

**I.4 Analysis of results**

Tumour formation rate is calculated according to equation (I1)

(I1)
Tumour formation rate=N/M×100%

In this equation: M—the total number of injected mice. N—the number of tumour‐bearing mice.
