# Supplementary material for: Human mesenchymal stem cells
Source: Cell Prolif. 2021 Dec 22;55(4):e13141. doi: 10.1111/cpr.13141 (PMC9055891; doi:10.1111/cpr.13141)
Supplement: Supplementary file 1 — Appendix A: Cell viability test (cell enumeration method) Appendix B: Detection of cell surface markers (Flow cytometry) Appendix C: Detection of induced IDO expression (PCR assay) Appendix D: T cell proliferation inhibition assay (CFSE assay) Appendix E: Inhibition assay of IFN‐γ and TNF‐α secretion by T cells (Intracellular Cytokine Staining, ICS) Appendix F: Osteogenic differentiation assay (Alizarin red S staining) Appendix G: Adipogenic differentiation assay (Oil red O staining) Appendix H: Chondrogenic differentiation assay (Alcian blue staining) Appendix I: In vivo Tumorigenicity Testing (immunodeficient mice method) [file CPR-55-e13141-s001.docx]

Appendix S1

**Appendix A**

**(Normative appendix)**

**Cell viability test (cell enumeration method)**

**A.1 Instruments**

A.1.1 Microscope.

A.1.2 Haemocytometer.

**A.2 Reagents**

Unless otherwise stated, all reagents used shall be analytical grade. The water used for testing shall be deionized water.

A.2.1 Phosphate buffered saline (PBS): pH 7.4.

A.2.2 Trypan blue solution: dilute to 0.4% (W/V) with phosphate buffered saline (A.2.1).

**A.3 Testing protocol**

A.3.1 Preparation of cell suspension

Harvest and suspend the cells with appropriate volume of Phosphate buffered saline (A.2.1). The cells in the haemocytometer shall be 20 ~ 50 cells/mm^2^. Serial dilution is necessary if the number of cells exceeds 200 per haemocytometer.

A.3.2 Trypan blue staining

Evenly mix the Trypan blue solution (A.2.2) with the cell suspension (A.3.1) at a volume ratio of 1:1.

A.3.3 Cell counting

Load the haemocytometer (A.1.2) with 10 μL of the trypan blue-labelled sample (A.3.2). Make sure the entire chamber is filled with the testing sample. Stand for 30 seconds, count the stained cells and the total number of cells, respectively.

For the 16 × 25 counting chamber, use the four 1 mm^2^ medium squares at the top left, top right, bottom left and bottom right of the chamber (ie 100 small squares) for counting. For the 25 × 16 counting chamber, use the five 1 mm^2^ medium squares at the top left, top right, bottom left, bottom right and centre of the chamber (ie 80 small squares) for counting. When there are cells on the lines of the large square, only cells on the top line and left line of the large square can be counted (or alternatively only cells on the bottom line and right line).

Repeat steps A.3.2 ~ A.3.3 for another sample.

A.3.4 Cell survival rate calculation

**A.4 Calculation and analysis**

Cell viability is calculated according to equation (A.1)

Cell viability = (M - S)/M × 100% (A.1)

In this equation:

M – Total number of cells

S – Number of stained cells

The viability of cells is the mean of two duplicate samples.

**A.5 Accuracy**

The absolute difference value between the two independent tests by the independent inter-examiner, under the same conditions, shall not exceed 10% of their arithmetic mean.

**Appendix B**

**(Normative appendix)**

**Detection of cell surface markers (Flow cytometry)**

**B.1 Instruments**

B.1.1 Flow cytometer.

B.1.2 Bench-top centrifuge.

B.1.3 Electronic balance.

**B.2 Reagents**

Unless otherwise stated, all the reagents used shall be analytical grade. The water used in the experiment shall be Grade 1 water as stipulated in GB/T 6682.

B.2.1 Phosphate buffered saline (PBS): pH7.4.

B.2.2 Bovine serum albumin (BSA): Purity ≥ 98%.

B.2.3 Sodium azide (NaN_3_).

B.2.4 Antibodies (including Anti-human CD105, CD73, CD90, CD11b, CD19, CD31, CD34, CD45, HLA-DR antibodies and isotype control antibodies).

Note 1 to entry: make sure all the antibodies work well according to the manufacturer’s instructions. Live or dead cell staining could be use where necessary.

B.2.5 Use electronic balance (B.1.3), prepare the following solutions according to the relative requirements for flow cytometry: wash solution, antibody dilution solution.

**B.3 Sample storage**

The wash solution and fixed samples shall be stored at 2 ~ 8 ℃. Antibodies shall be stored according to the manufacturer’s instructions.

**B.4 Testing protocol**

B.4.1 Sample preparation

Collect samples by centrifuging single cell suspensions with Bench-top centrifuge (B.1.2) at 300 g for 4 min, and discard the supernatant. Wash the cell samples with an appropriate volume of wash solution, then collect samples by centrifuging at 300 g for 4 min, and discard the supernatant.

B.4.2 Antibody incubation

Incubate the samples with the diluted antibodies according to the manufacturer’s instructions. Wash the cell samples with an appropriate volume of wash solution for 2 times then centrifuge at 300 g for 4 min, and discard the supernatant.

B.4.3 Filter and detection

Resuspend the samples with wash solution and then transfer the cell suspension into flow cytometry tube passed through a 40 µm mesh filter. Load the samples into the flow cytometer and perform detection according to the manufacturer’s instruction.

B.4.4 Gating

Gate the population of target cells based on particle size and granularity, excluding cell debris and other irrelevant particles. The gating of positive staining cells shall be determined by the fluorescence intensity using isotype controls as a reference. Both positive and negative experimental controls shall be set up for gating and the following analysis.

**B.5 Analysis of results**

Analyse the results using software according to manufacturer’s instructions.

**Appendix C**

**(Normative appendix)**

**Detection of induced IDO expression (PCR assay)**

**C.1 Instruments**

C.1.1 Nucleic acid quantification machine.

C.1.2 PCR-Cycler.

C.1.3 Electrophoresis apparatus

C.1.4 Gel imager

**C.2 Reagents**

Unless otherwise stated, all the reagents used shall be analytical grade. The water used in the experiment shall be Grade 1 water as stipulated in GB/T 6682.

C.2.1 Human recombinant IFN-γ and human recombinant TNF-α.

C.2.2 RNA extraction kit.

C.2.3 Reverse transcription kit.

C.2.4 PCR primers.

C.2.5 Taq DNA polymerase.

C.2.6 Ladders.

**C.3 Testing protocol**

C.3.1 Treat the hMSC with IFN-γ or IFN-γ + TNF-α

Calculate the living cell concentration of hMSC suspension according to the method in Appendix A. Seed hMSC at a density of 1×10^5^ ~ 4×10^5^ cells/cm^2^, then culture with IFN-γ (10 ~ 30 ng/mL) or IFN-γ (10 ~ 30 ng/mL) + TNF-α (10 ~ 30 ng/mL), for 12 ~ 36 hours. At the same time, hMSC culture without IFN-γ or IFN-γ+TNF-α is set up as the control group and cultured for the same time.

C.3.2 Extraction of cellular RNA

Perform RNA extraction according to the manufacturer’s instructions, and use a Nucleic acid quantification machine (C.1.1) for nucleic acid content determination.

C.3.3 Reverse transcription polymerase chain reaction (RT-PCR)

Perform reverse transcription to acquire cDNA and amplify the gene of IDO via PCR-Cycler (C.1.2) according to the manufacturer’s instructions.

C.3.4 Electrophoresis of PCR products

Use electrophoresis apparatus (C.1.3) for electrophoresis testing that shall be performed according to the manufacturer’s instructions.

C.3.5 Gel imaging.

Perform the Gel imaging via Gel imager (C.1.4) according to the manufacturer’s instructions.

**C.4 Analysis of results**

The expression of IDO is detected in hMSC cultured with IFN-γ or IFN-γ + TNF-α, while it is undetectable in hMSC without stimulation.

**Appendix D**

**(Normative appendix)**

**T cell proliferation inhibition assay (CFSE assay)**

**D.1 Instruments**

D.1.1 Haemocytometer.

D.1.2 Microscope.

D.1.3 Bench-top centrifuge.

D.1.4 Flow cytometer.

**D.2 Reagents**

Unless otherwise stated, all the reagents used shall be analytical grade. The water used in the experiment shall be Grade 1 water as stipulated in GB/T 6682.

D.2.1 Phosphate buffered saline (PBS): pH7.4.

D.2.2 Cell dissociation enzyme.

D.2.3 Trypan Blue Solution: dilute to 0.4% (W/V) with phosphate buffered saline (D.2.1).

D.2.4 Phytohaemagglutinin, PHA

D.2.5 Leukocyte separation solution (Ficoll)

D.2.6 Antibodies (such as anti-CD3 antibody)

D.2.7 Carboxyfluorescein diacetate succinimidyl ester (CFSE)

**D.3 Testing protocol**

D.3.1 T cell separation and staining

D.3.1.1 T cell separation

Use Ficoll (D.2.5) to separate peripheral blood mononuclear cells (PBMC), and wash twice with an appropriate volume of sterile phosphate buffer saline (D.2.1) by bench-top centrifuge (D.1.3). Incubate the samples with the diluted antibodies and then wash twice with an appropriate volume of sterile phosphate buffer saline (D.2.1) by bench-top centrifuge (D.1.3). Resuspend the samples and dilute the cell suspension to 5 × 10^7^ cells/mL with wash solution and then transfer the cell suspension into flow cytometry tube passed through a 40 µm mesh filter. Load the samples into the flow cytometer (D.1.4) and perform cell sorting according to the manufacturer’s instructions.

D.3.1.2 T cell staining

Use haemocytometer (D.1.1) and microscope (D.1.2) to calculate the living cell concentration of T cells according to the method in Appendix A. Label T cells with CFSE according to the manufacturer’s instructions.

D.3.2 Co-culture T cells with hMSC

D.3.2.1 T cell proliferation

Use haemocytometer (D.1.1) and microscope (D.1.2) to calculate the living cell concentration of CFSE-labelled T cells suspension according to the method in Appendix A. Seed the T cells at a density of 1×10^6^ cells/cm^2^, and stimulate with 2 ~ 5 µg/mL PHA (D.2.4). T cell cultures without PHA are set up as the control group. After 96 hours culture, the percentage of T cell proliferation after stimulation is detected and record it as A.

D.3.2.2 Suppression of T cell proliferation by hMSC

Use cell dissociation enzyme to dissociate hMSC and prepare as single cell suspension. Use haemocytometer (D.1.1) and microscope (D.1.2) to calculate the living cell concentration of hMSC suspension according to the method in Appendix A. Seed the hMSC at a density of 2 × 10^5^ cells/cm^2^. Then co-culture with CFSE-labelled T cells at a ratio of 5:1 (T cells: hMSC) in the presence of 2 ~ 5 µg/mL PHA (D.2.4). After 96 hours culture, the percentage of T cell proliferation that cultured with hMSC is detected and record it as C.

D.3.3 Collection and detection of T cells

Collect the T cells (D.3.2), and wash T cells samples twice with sterile phosphate buffer saline (D.2.1) by bench-top centrifuge (D.1.3), then transfer the cell suspension into flow cytometry tube passed through a 40 µm mesh filter. Load the samples into the flow cytometer (D.1.4) and perform detection according to the manufacturer’s instructions.

D.3.4 Gating

Gate the population 1 of target cells based on particle size and granularity, excluding cell debris and other irrelevant particles. Then, gate the parent population (zeroth generation) in population1 according to the fluorescence intensity of T cells without PHA stimulation, and gate the proliferating cells assigned as population 2 (the percentage of T cell proliferation) based on the position of parent population.

**D.4 Analysis of results**

Analyse the results using software according to manufacturer’s instructions and calculate the inhibition rate of hMSC on T cell proliferation.

Inhibition rate is calculated according to equation (D.1):

Inhibition rate = (A - C)/A × 100% (D.1)

In this equation:

A – The percentage of the proliferating T cells without hMSC

C –The percentage of the proliferating T cells that cultured with hMSC

**Appendix E**

**(Normative appendix)**

**Inhibition assay of IFN-γ and TNF-α secretion by T cells**

**(Intracellular Cytokine Staining, ICS)**

**E.1 Instruments**

E.1.1 Haemocytometer.

E.1.2 Microscope.

E.1.3 Bench-top centrifuge.

E.1.4 Flow cytometer.

**E.2 Reagents**

Unless otherwise stated, all the reagents used shall be analytical grade. The water used in the experiment shall be Grade 1 water as stipulated in GB/T 6682.

E.2.1 Phosphate buffered saline (PBS): pH7.4.

E.2.2 Cell dissociation enzyme.

E.2.3 Trypan Blue Solution: dilute to 0.4% (W/V) with phosphate buffered saline (E.2.1).

E.2.4 Phorbol ester (PMA).

E.2.5 Leukocyte separation solution (Ficoll).

E.2.6 Antibodies (such as anti-CD3, anti-IFN-γ and anti-TNF-α antibodies).

E.2.7 Brefeldin A (BFA).

E.2.8 Ionomycin.

E.2.9 Saponin.

E.2.10 4% PFA.

**E.3 Testing protocol**

E.3.1 T cell separation

Use Ficoll (E.2.5) to separate peripheral blood mononuclear cells (PBMC), and wash twice with an appropriate volume of sterile phosphate buffer saline (E.2.1) by bench-top centrifuge (E.1.3). Incubate the PBMC with the diluted antibodies (E.2.6) and then wash twice with an appropriate volume of sterile phosphate buffer saline (H.2.1). Resuspend the PBMC and dilute the cell suspension to 5 × 10^7^ cells/mL with wash solution and then transfer the cell suspension into flow cytometry tube passed through a 40 µm mesh filter. Load the samples into the flow cytometer (E.1.4) and perform T cells sorting according to the manufacturer’s instructions.

E.3.2 Co-culturing of T cells with hMSC

E.3.2.1 T cell inflammatory factor secretion

Use haemocytometer (E.1.1) and microscope (E.1.2) to calculate the living cell concentration of T cells suspension according to the method in Appendix A. Seed T cells at a density of 1×10^6^ cells/cm^2^, and culture for 48 hours. Add 50 ng/mL PMA (E.2.4), 1 μg/mL Ionomycin (E.2.8) and 10 μg/mL BFA (E.2.7) to the culture system 4 ~ 6 hours before the end of the culture. Detect the proportion of IFN-γ^+^ T cells and record as A1, and the proportion of TNF-α^+^T cells and record as A2.

E.3.2.2 Suppression T cell cytokine secretion by hMSC

Use cell dissociation enzyme to dissociate hMSC and prepare as single cell suspension. Use haemocytometer (E.1.1) and microscope (E.1.2) to calculate the living cell concentration of h hMSC and T cells suspension according to the method in Appendix A. Seed hMSC at a density of 2×10^5^ cells/cm^2^. Then co-culture with T cells at a ratio of 5:1 (T cells: hMSC). Culture for 48 hours, and add 50 ng/mL PMA (E.2.4), 1 μg/mL Ionomycin (E.2.8), and 10 μg/mL BFA (E.2.7) to the culture system 4 ~ 6 hours before the end of the culture. Detect the proportion of IFN-γ^+^ T cells in the T/hMSC culture system and record as C1, and the proportion of TNF-α^+^T cells in the T/hMSC culture system and record as C2.

E.3.3 Collection and detection of T cells

Collect the T cells (E.3.2), and wash T cells samples twice with sterile phosphate buffer saline (E.2.1) and fix cells with 4% PFA (E.2.10), then wash T cells samples twice with sterile phosphate buffer saline (E.2.1), and treat cells with 0.1% ~ 0.2% saponin (E.2.9). Incubate the samples with the diluted antibodies and wash T cells samples with sterile phosphate buffer saline (E.2.1), and transfer the cell suspension into flow cytometry tube passed through a 40 µm mesh filter. Load the samples into the flow cytometer (E.1.4) and perform detecting according to the manufacturer’s instructions.

E.3.4 Gating

Gate the population 1 of target cells based on particle size and granularity, excluding cell debris and other irrelevant particles. According to the fluorescence intensity of the isotype control, gate the cell population of IFN-γ^+^ T cells within population 1, as well as the cell population of TNF-α^+^ T cells, excluding negative cells that are not labelled by fluorescent antibodies.

**E.4 Analysis of results**

Analyse the results using software according to manufacturer’s instructions and calculate the inhibition rate of hMSC on the IFN-γ and TNF-α secretion of T cells.

Inhibition rate is calculated according to equation (E.1) and (E.2):

IFN-γ inhibition rate = (A1 – C1)/A1 × 100% (E.1)

In this equation:

A1 – the percentage of IFN-γ^+^ T cells without hMSC.

C1 – the percentage of IFN-γ^+^ T cells that cultured with hMSC.

TNF-α inhibition rate = (A2– C2)/A2 × 100% (E.2)

In this equation:

A2 –the percentage of TNF-α^+^ T cells without hMSC.

C2 – the percentage of TNF-α^+^ T cells that cultured with hMSC.

**Appendix F**

**(Normative appendix)**

**Osteogenic differentiation assay (Alizarin red S staining)**

**F.1 Instruments**

F.1.1 Haemocytometer.

F.1.2 Microscope.

F.1.3 Bench-top centrifuge.

**F.2 Reagents**

Unless otherwise stated, all the reagents used shall be analytical grade. The water used in the experiment shall be Grade 1 water as stipulated in GB/T 6682.

F.2.1 Phosphate buffered saline (PBS): pH7.4.

F.2.2 Cell dissociation enzyme.

F.2.3 Trypan Blue Solution: dilute to 0.4% (W/V) with phosphate buffered saline (F.2.1).

F.2.4 Osteogenic differentiation medium.

F.2.5 Alizarin red S staining kit.

**F.3 Testing protocol**

F.3.1 Sample preparation

F.3.1.1 Cell dissociation

Use cell dissociation enzyme to dissociate hMSC, collect hMSC by bench-top centrifuge (F.1.3), and gently resuspend the cells in saline to avoid the formation of bubbles or residual cell clumps.

F.3.1.2 Cell counting

Use haemocytometer (F.1.1) and microscope (F.1.2) to calculate the live cell concentration of the cell suspension, according to the method in Appendix A.

F.3.2 Cell seeding and induction

Choose the appropriate cell seeding method, seeding density and induction procedure according to the manufacturer’s instructions and osteogenic differentiation is induced for 14 ~ 21 days.

F.3.3 Calcium deposits staining

Stain the extracellular calcium deposits by Alizarin Red S staining according to the manufacturer’s instructions.

**F.4 Analysis of results**

A large number of bright orange-red calcium deposits can be seen under the microscope.

**Appendix G**

**(Normative appendix)**

**Adipogenic differentiation assay (Oil red O staining)**

**G.1** **Instruments**

G.1.1 Haemocytometer.

G.1.2 Microscope.

G.1.3 Bench-top centrifuge.

**G.2 Reagents**

Unless otherwise stated, all the reagents used shall be analytical grade. The water used in the experiment shall be Grade 1 water as stipulated in GB/T 6682.

G.2.1 Phosphate buffered saline (PBS): pH7.4.

G.2.2 Cell dissociation enzyme.

G.2.3 Trypan Blue Solution: dilute to 0.4% (W/V) with phosphate buffered saline (G.2.1).

G.2.4 Adipogenic differentiation medium.

G.2.5 Oil red O staining kit.

**G.3 Testing protocol**

G.3.1 Sample preparation

G.3.1.1 Cell dissociation

Use cell dissociation enzyme to dissociate hMSC, collect hMSC by bench-top centrifuge (G.1.3), and gently resuspend the cells in saline to avoid the formation of bubbles or residual cell clumps.

G.3.1.2 Cell counting

Use haemocytometer (G.1.1) and microscope (G.1.2) to calculate the live cell concentration of the cell suspension, according to the method in Appendix A.

G.3.2 Cell seeding and induction

Choose the appropriate cell seeding method, seeding density and induction procedure according to the manufacturer’s instructions and adipogenic differentiation is induced for 14 ~ 21 days.

G.3.3 Lipid droplet staining

Stain the lipid droplets by Oil red O according to the manufacturer’s instructions.

**G.4 Analysis of results**

Orange-red lipid droplets can be seen under the microscope, and fat cells contain lipid droplets of varying sizes.

**Appendix H**

**(Normative appendix)**

**Chondrogenic differentiation assay (****Alcian blue staining)**

**H.1 Instruments**

H.1.1 Haemocytometer.

H.1.2 Microscope.

H.1.3 Bench-top centrifuge.

**H.2 Reagents**

Unless otherwise stated, all the reagents used shall be analytical grade. The water used in the experiment shall be Grade 1 water as stipulated in GB/T 6682.

H.2.1 Phosphate buffered saline (PBS): pH7.4.

H.2.2 Cell dissociation enzyme.

H.2.3 Trypan Blue Solution: dilute to 0.4% (W/V) with phosphate buffered saline (H.2.1).

H.2.4 Chondrogenic differentiation medium.

H.2.5 Alcian blue staining kit.

**H.3** **Testing protocol**

H.3.1 Sample preparation

H.3.1.1 Cell dissociation

Use cell dissociation enzyme to dissociate hMSC, collect hMSC by bench-top centrifuge (H.1.3), and gently resuspend the cells in saline to avoid the formation of bubbles or residual cell clumps.

H.3.1.2 Cell counting

Use haemocytometer (H.1.1) and microscope (H.1.2) to calculate the live cell concentration of the cell suspension, according to the method in Appendix A.

H.3.2 Cell seeding and induction

Choose the appropriate cell seeding method, seeding density and induction procedure according to the manufacturer’s instructions and chondrogenic differentiation is induced for 14 ~ 21 days.

H.3.3 Cartilage extracellular matrix staining

Stain the cartilage extracellular matrix by Alcian blue according to the manufacturer’s instructions.

**H.4 Analysis of results**

The dark blue extracellular matrix of chondrocytes can be seen under the microscope.

**Appendix I**

**(Normative appendix)**

**In vivo Tumorigenicity** **Testing (immunodeficient mice method)**

**I.1 Instruments**

I.1.1 Haemocytometer.

I.1.2 Microscope.

I.1.3 Bench-top centrifuge.

**I.2 Reagents**

Unless otherwise stated, all the reagents used shall be analytical grade. The water used in the experiment shall be Grade 1 water as stipulated in GB/T 6682.

I.2.1 Phosphate buffered saline (PBS): pH7.4.

I.2.2 Cell dissociation enzyme.

I.2.3 Trypan Blue Solution: dilute to 0.4% (W/V) with phosphate buffered saline (I.2.1).

**I.3 Testing protocol**

I.3.1 Sample preparation

I.3.1.1 Cell dissociation

Use cell dissociation enzyme to dissociate hMSC, collect hMSC by bench-top centrifuge (I.1.3) and gently resuspend the cells in saline to avoid the formation of bubbles or residual cell clumps.

I.3.1.2 Cell counting

Use haemocytometer (I.1.1) and microscope (I.1.2) to calculate the live cell concentration of the cell suspension, according to the method in Appendix A.

I.3.2 Cell transplantation

1×10^7^ hMSC are injected subcutaneously into immunodeficient mice aged 6 to 8 weeks. Set up a blank control group (injected with the solvent corresponding to the product), a negative control group (human diploid cells) and a positive control group (human tumour cell lines, injected according to the number of tumour cell line inoculation requirements).

I.3.3 Tumour observation

Observe 16 weeks after injection, and measure body weight and tumour size every week. If the tumour in the tumour-bearing mice exceeded 2000 mm^3^, the tumour was ulcerated or severe weight loss, the humane endpoint could be considered. Remove the tumour from the mice, and perform gross observation. Then weigh the tumour and calculate the tumour formation rate.

**I.4 Analysis of results**

Tumour formation rate is calculated according to equation (I.1)

Tumour formation rate = N/M × 100% (I.1)

In this equation:

M – The total number of injected mice

N – The number of tumour-bearing mice
